# Effect of Decellularization Protocol on the Mechanical Behavior of Porcine Descending Aorta

**DOI:** 10.1155/2010/620503

**Published:** 2010-07-04

**Authors:** John C. Fitzpatrick, Peter M. Clark, Franco M. Capaldi

**Affiliations:** ^1^Department of Mechanical Engineering and Mechanics, Drexel University, 115 Randell Hall, 3141 Chestnut St., Philadelphia, PA 19104, USA; ^2^School of Biomedical Engineering, Science and Health Systems, Drexel University, 3141 Chestnut St., Philadelphia, PA 19104, USA

## Abstract

Enzymatic-detergent decellularization treatments may use a combination of chemical reagents to reduce vascular tissue to sterilized scaffolds, which may be seeded with endothelial cells and implanted with a low risk of rejection. However, these chemicals may alter the mechanical properties of the native tissue and contribute to graft compliance mismatch. Uniaxial tensile data obtained from native and decellularized longitudinal aortic tissue samples was analyzed in terms of engineering stress and fit to a modified form of the Yeoh rubber model. One decellularization protocol used SDS, while the other two used TritonX-100, RNase-A, and DNase-I in combination with EDTA or sodium-deoxycholate. Statistical significance of Yeoh model parameters was determined by paired *t*-test analysis. The TritonX-100/EDTA and 0.075% SDS treatments resulted in relatively variable mechanical changes and did not effectively lyse VSMCs in aortic tissue. The TritonX-100/sodium-deoxycholate treatment effectively lysed VSMCs and was characterized by less variability in mechanical behavior. The data suggests a TritonX-100/sodium-deoxycholate treatment is a more effective option than TritonX-100/EDTA and SDS treatments for the preparation of aortic xenografts and allografts because it effectively lyses VSMCs and is the least likely treatment, among those considered, to promote a decrease in mechanical compliance.

## 1. Introduction

In the United States, there are over 500,000 arterial bypass operations performed each year [1–3]. Autologous vessels are preferred as graft materials, however, up to 40% of patients needing bypass surgery may not have a healthy artery or saphenous vein of suitable length for use as an autograft [4]. Even if suitable venous tissue is available for transplantation, *in vivo* remodeling due to injury or increased loading may cause occlusion of the vessel [5, 6]. While synthetic grafts are currently the standard clinical alternative to autologous grafts, there are relative differences in mechanical properties between grafts formed from these materials and the native vasculature. As a result, the patient's native artery will biologically remodel itself to compensate for this mechanical difference and excessive remodeling may occlude the artery. This excessive thickening, called intimal hyperplasia, is the main mode of occlusive graft failure and results in low long-term patency rates for small-diameter arterial grafts.

Synthetic grafts such as Dacron (polyethylene terephthalate) and expanded polytetraflouroethylene (ePTFE) function exceptionally well under high flow, low-resistance conditions. Both Dacron and ePTFE have had great success in replacing large diameter vessels, maintaining a reported 90% patency rate after five-years as an aortiobifemoral graft [7]. While ePTFE grafts have been commercially available for transplant for over 40 years, these grafts have a 20% decreased patency rate over a five year period when compared with autologous saphenous vein grafts for femoropopliteal bypass procedures [8], and are known to elicit thrombotic complications when used for peripheral bypass applications, effectively decreasing their long term patency [9, 10]. 

In light of the complications associated with small-diameter synthetic grafts, there have been efforts to create a tissue-engineered blood vessel *in vitro*, but these vessels are susceptible to rupture and are not a timely solution [11–15]. Recent studies on arterial allografts [16–18] and xenografts [14, 19–21] have shown that an arterial extracellular matrix may be preserved and seeded with the host's endothelial cells to prevent immunogenic rejection. These processes are advantageous because the graft is available in days rather than weeks or months. Xenogenic extracellular matrices isolated from various tissues of *sus scrofa domesticus*, the common domesticated pig, have been used as a scaffold for a variety of tissue engineering applications including lower urinary tract reconstruction, skin reconstruction, orthopaedic applications, and arterial grafts [22]. Acellular xenogeneic scaffolds have the unique potential for host cell recolonization without inducing an immunogenic response [23], since the amino acid sequence and quaternary structure of many extracellular matrix proteins are highly conserved among various species [22, 24]. The availability of suitable synthetic, allogeneic, or xenogenic graft scaffolds for small-diameter arteries would greatly aid patients needing coronary or peripheral bypass surgery. 

The goal of any decellularization procedure is to remove all of the cellular components from the extracellular matrix while preserving its biological activity and mechanical integrity. Decellularization protocols may involve any combination of physical, chemical or enzymatic methods and utilize a variety of detergents, enzymes or solvents to lyse the native smooth muscle cells (VSMCs) from the extracellular matrix [24]. The treatments effectively reduce antigenicity while creating free volume spaces upon which the host's native cells are able to proliferate [25, 26]. 

Among the various decellularization procedures in literature, we compared the effects of three well-established enzymatic-and-detergent treatments [25, 27, 28]. One of the tested protocols uses SDS as the sole reagent, while the remaining two use RNase A, DNase I, and TritonX-100 in combination with EDTA or sodium-deoxycholate (the salt of deoxycholic acid). The selected protocols have been used to successfully decellularize porcine heart valve roots and leaflets [25, 27, 29–36] but will be extended to fully intact porcine arterial tissue segments for the first time in this study. 

The selected TritonX-100/EDTA treatment was used successfully as a decellularization method in heart valve decellularization-recellularization studies [27, 29] but was then used without RNase when decellularizing porcine artery without its adventitial layer by Dahl et al. [37] who found no significant difference in DNA content between treated and untreated vessels. Booth et al. championed 0.03%–1% SDS and 0.5%–2% deoxycholic acid treatments as successful decellularization techniques for porcine heart valves [31]. Since then, Reider et al. reported difficulty with recellularizing vessels after decellularization in 0.1% SDS solution and Kasimir et al. also noticed disintegration of matrix fibers during SEM analysis following a 0.03% SDS treatment [33, 34]. Deoxycholic acid treatments have had more reported success than SDS treatments recently, as Erdbrügger et al. successfully implanted porcine pulmonary valves in juvenile sheep and human models after a 1% deoxycholic acid treatment. Likewise, Fang et al. successfully decellularized porcine aortic valves with a protocol similar to the selected TritonX-100/sodium-deoxycholate treatment and subsequently recellularized the valves with endothelial progenitor cells derived from human umbilical cord [35, 36]. Deoxycholic acid treatments also show promise from a mechanical standpoint, as a 4% deoxycholic acid solution was used to decellularize canine common carotid artery without significant changes in compliance between native and decellularized vessels [38].

 Though decellularization procedures produce acellular matrices that can be re-endothelialized with a host's autologous cells to minimize thrombosis when implanted as a graft, these treatments have been shown to change the mechanical compliance of arterial tissue [26, 37]. An engineered graft must not only remain biocompatible, but it must also exhibit mechanical properties similar to the native vasculature which it will replace [39, 40]. Differing mechanical behavior between the graft and native vasculature is known as compliance mismatch. This condition creates local flow disturbances, which may lower the vessel wall shear stress at the graft anastomosis and lead to the progressive development of intimal hyperplasia, the most common cause of occlusive graft failure [41–47].

To date, there have been several studies that have investigated the mechanical effects of decellularization on vascular tissue segments [16, 20, 28, 37, 38, 48]. Some studies compare vessel compliances under inflation loadings [38, 48]. Other studies compare rupture properties of native and acellular vessels, but do not quantify differences observed in the 1.3 ≤ *λ*_*z*_ ≤ 1.6 axial stretch range of load-displacement curves [20, 28, 37], even though this is generally accepted as the *in vivo* stretch range [49, 50]. These studies may also have shortcomings in testing methodology that affect reported results.

One previous study compared native and acellular porcine carotid artery lengths using inflation tests, but the adventitial layer was removed to expedite the decellularization process [37]. The effect of decellularization on the adventitia cannot be ignored, since certain detergents such as SDS or deoxycholic acid may disrupt structural protein-protein interactions [24]. Among the xenograft and allograft studies previously mentioned, [20, 37] compared native and acellular samples that had not been preconditioned, even though results obtained from preconditioned soft tissue samples are typically reported in order to minimize the effects of hysteresis [51]. The mechanical environment of arteries was not fully considered in a study that compared native and acellular arteries using compression testing [16]; because arterial tissue is in a constant physiological state of tension due to systolic and diastolic pressures, arterial tissue should be tested for changes in its tensile properties. Though inflation testing is the current standard for defining anisotropic parameters for constitutive models of blood vessels [37, 50, 52, 53], previous investigators report that longitudinal strip samples of arterial tissue display characteristic hyperelastic behavior [54–58]. This is to be expected, since collagen and elastin fibers are partially oriented in the longitudinal direction.

Similar to arterial tissue, rubber exhibits nonlinear, hyperelastic behavior at large strains. A parallel between these materials' behavior is the existence of chain-like molecular structures; rubber is composed of polymer chains, and each arterial layer has oriented fibers [54]. Recently, Martins et al. investigated the applicability of fitting uniaxial tensile data for silicone rubber and biological tissue to popular material models [59]. They found that Yeoh, Ogden, and Martins models fit both sets of data with correlation coefficients higher than *R* = 0.99, which are considered very good fits. Martins et al. note that the Ogden and Martins models are based on exponential functions, while the Yeoh model is a general hyperelastic function which can fit a wide range of materials. The present investigation seeks to improve upon the aforementioned biomechanical studies by using paired samples of (i) intact vessel strips (ii) cut from the same (longitudinal) location of an artery, wherein (iii) both samples have identical stress histories, and (iv) the mechanical data is quantified in terms of the entire stress curve through a portion of the physiological stretch range. 

## 2. Materials and Methods

Freshly excised segments of porcine aorta were trimmed and sectioned longitudinally to make sets of paired samples. Of these pairs, one sample was tested as a control (native) and the other (acellular) underwent a decellularization procedure. Multiple aortas were used in each protocol's data set, as shown in Table 1.

### 2.1. Decellularization Protocols

Protocol A uses a solution of 1% w/w tert-octylphenyl-polyoxythylen (Triton X-100; Fisher Scientific, Fair Lawn, NJ) and 0.02% w/w ethylenediaminetetraacetic acid (EDTA; Fisher Scientific Fair Lawn, NJ), in Phosphate Buffered Saline solution (PBS; MA031, Aqua Solutions, Deer Park, TX). Samples marked for decellularization were constantly agitated in this solution for 24 hours at 37°C with RNase A (20 *μ*g/mL; Roche Diagnostics, Mannheim, Germany) and DNase I (0.2 mg/mL; Roche Diagnostics, Mannheim, Germany) before several rinse cycles in PBS [27]. A minimal change is expected with Protocol A, since Triton X-100 will disrupt lipid-lipid and lipid-protein interactions while EDTA removes cellular material by binding to divalent cations at cellular adhesion integrins [24]. 

As specified in Schaner et al. [60], Protocol B uses 0.075% w/w sodium dodecyl sulfate (SDS; MP Biomedicals, Solon, OH) in PBS. Samples marked for decellularization were constantly agitated in this solution for 15 hours at 37°C before five 15-minute rinse cycles in PBS at 37°C. Protocol B may induce a moderate change in the mechanical properties of the tissue, as the hydrophobic-hydrophilic and ionic properties of SDS can alter protein-protein interactions by opening the molecular structure of elastin and disrupting the hydrogen bonding of collagen [61]. 

The final protocol tested, Protocol C, uses 0.25% w/w Triton X-100 and 0.25% w/w sodium-deoxycholate (Fisher Scientific, Fair Lawn, NJ) in PBS. Samples marked for decellularization were constantly agitated in solution for 24 hours at 37°C before a 72-hour wash cycle in M-199 medium (Mediatech, Inc., Herndon, VA) kept at 4°C. Following the wash cycle, the marked samples were treated with RNase A (100 *μ*g/mL) and DNase I (150 IU/mL) with 50 mmol MgCl_2_ (Fisher Scientific, Fair Lawn, NJ) in PBS for 24 hours at 37°C. After nuclease digestion, samples were again washed with M-199 medium for 24 hours at 4°C [34]. According to a report comparing detergents used for decellularization protocols, Protocol C may cause moderate to severe changes in mechanical properties, since deoxycholic acid is regarded as a more disruptive ionic detergent than SDS [24]. 

The same types of PBS, Triton X-100, RNase A, and DNase I were used for applicable protocols throughout the study.

### 2.2. Histological Methods

Three histological sections were taken from different sections of proximal (*d*_outer_≈ 12–15 mm) and distal (*d*_outer_≈ 10–12 mm) acellular arterial samples in each decellularization run, and compared to native tissue in order to verify tissue decellularization. Mechanical testing was not performed on the native and acellular samples from which these histological sections were taken. Samples were transferred to a 30% by weight sucrose/PBS solution, embedded in Tissue-Tek OCT (Sakura Finetek, Torrance, CA), and sectioned on a Reichert Jung 2800 Frigocut-E cryostat after sucrose saturation. Sections with 15–20 *μ*m thickness were transferred to slides and stained with a Mayer's Hematoxylin and Eosin stain kit (SL029; DakoCytomation, Carpinteria, CA). The stained sections were then examined under a light microscope for the presence of vascular smooth muscle cell nuclei. 

### 2.3. Paired Sample Testing

**Figure 1:**
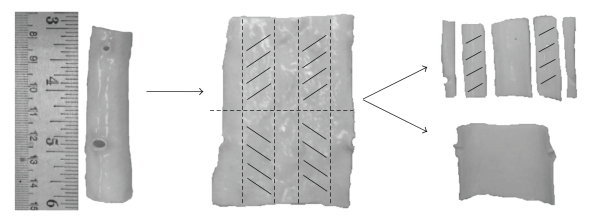
Longitudinal sample preparation: cuts made on dashed lines, similarly cross-hatched sections are paired samples.

Excised portions of aortic vessels from adult pigs weighing 90–105 kg were obtained from an abbatoir (Kolb Brothers, Spring City, PA) and transported in a 0.9% saline solution on ice for no more than two hours. A 150 mm aortic length that tapered in external diameter from 15 mm to 10 mm was removed from the artery. After being trimmed of connective tissue, the vessel was cut with surgical scissors along its line of ventricles, pressed flat, and sectioned every 30 mm. Paired, similar 10 mm-wide longitudinal (axial) samples were made from these sections (Figure 1). Each set of arterial samples designated for decellularization was tested immediately after the designated decellularization incubation periods specific to each protocol. 

#### 2.3.1. Mechanical Testing Procedures

Prior to testing, the thickness and width of each longitudinal artery sample was measured at three points with a digital caliper. These measurements were averaged and used to calculate each sample's cross-sectional area, *A*_avg_. The error due to variations in thickness and width of the samples is on the order of 10%, but does not affect the conclusion of this study since the changes in mechanical behavior between native and decellularized samples for all considered protocols is greater than the range of error. 

**Figure 2:**
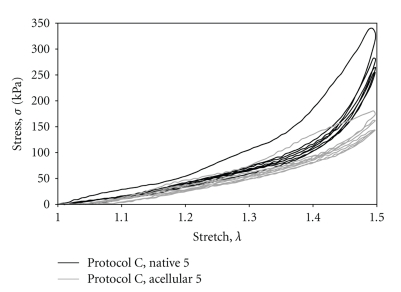
Example of hysteresis in native and acellular tissue samples for 4 load-unload cycles.

Each tensile specimen was loaded into notched stainless-steel grips set 20 mm apart. Digital caliper readings were taken to determine the actual gage length in zero-stress state. The sample was then immersed in an isotonic (154 mmol NaCl) saline bath within a plexiglas cylinder affixed to the base. The temperature of the bath was maintained at 38°C for 15 minutes prior to tensile loading. A force-displacement curve was recorded at a strain rate of 0.1 mm/s (1%/s) for three preconditioning load-unload cycles and a final, fourth load-unload cycle that was analyzed. The four load-unload hysteresis loops resulting from preconditioning and the analyzed data set are shown in Figure 2, wherein the third and fourth loops overlap significantly. Each loading cycle ended at a longitudinal stretch ratio of approximately 1.50. The stress concentration within the tissue near the serrated, mated grips caused failure (tearing) at stretch ratios higher than 1.50. 

**Figure 3:**
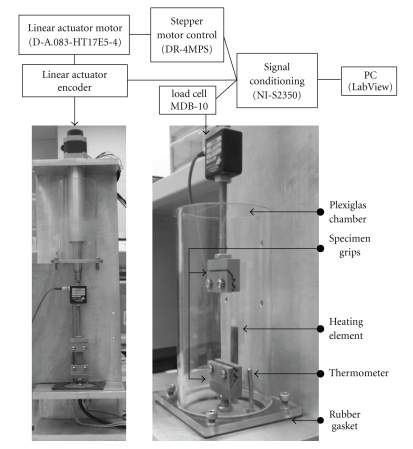
Uniaxial tensile system components and controllers.

The experimental uniaxial tensile system (Figure 3) consists of a linear actuator (D-A.083-HT17E5-4-1NO-/4; Ultra Motion, Cutchogue, NY) controlled by a stepper motor outfitted with optically isolated inputs (DR-4MPS; Advanced Micro Systems, Nashua, NH). A signal conditioning controller (SC-2350; National Instruments, Austin, TX) was configured to output step signals to a stepper motor while acquiring data from the linear actuator's encoder and a load cell (MDB-10; Transducer Techniques, Temecula, CA). The accuracy of the load cell, calculated from uncertainties in terms of its percentage of rated output, is within 0.2870 N for all experimental measurements taken. The linear actuator was mounted in an inverted position and threaded rod lengths connected the grips to the load cell and base of the apparatus. The linear actuator encoder and load cell were configured as DAQ inputs in a LabVIEW VI application (National Instruments, Austin, TX).

#### 2.3.2. Mechanical Data Analysis

Taking an approach similar to Martins et al. [59], uniaxial data sets were fit to several rubber models and the variation of coefficients between paired native and acellular strip samples was considered. Yeoh parameters varied the least among the material models. For this purpose the Yeoh model is used to assess changes in mechanical properties between native and decellularized porcine aorta strips [62]. The stress states of the paired samples before decellularization are assumed to be equal, and axial stretch (*λ*) is computed from the reference length of the vessel in the zero-stress state. The stress was computed as engineering stress, which is the experimental force acting on the plane of the cross-sectional area in the reference configuration,

(1) $$\sigma_{\text{experimental}}=\frac{F_{\text{experimental}}}{A_{0,\text{reference}}}.$$

In order to minimize the effects of hysteresis in each tissue sample, only the final (fourth) loading curve was analyzed. The stress-stretch data was normalized for stretch values between 1.00 and 1.50. Using a least-squares analysis in Excel, the Yeoh model function

(2) $$\sigma_{\text{Yeoh}}=2\left(\lambda^{2}-\frac{1}{\lambda }\right)\left(c_{1}+c_{2}\left(I_{1}-3\right)+c_{3}{\left(I_{1}-3\right)}^{2}\right)$$

was used to fit the data, where

(3) $$I_{1}=\lambda^{2}+\frac{2}{\lambda },$$

and *λ* is the stretch ratio, *I_1_* is the trace of the Right Cauchy-Green tensor, and *c*_1_, *c*_2_, and *c*_3_ are fitting constants defined in a least-squares analysis [59]. While fitting the Yeoh model to the data, there was no significant change (Δ*R* < 0.006) in the quality of the fit if *c*_2_ was excluded, so only the linear Hookean term c_1 _ and the strain-hardening “hyperelastic” term *c*_3_ were considered to simplify the analysis. Curve fit constants were determined by fitting experimental data to the modified Yeoh equation

(4) $$\sigma_{\text{Yeoh}}=2\left(\lambda^{2}-\frac{1}{\lambda }\right)\left(c_{1}+c_{3}{\left(I_{1}-3\right)}^{2}\right).$$

Results from sample pairs whose curve fits can be visually identified as inaccurate fits or whose correlation coefficients (*R*) are less than 0.992 have not been considered. The correlation coefficient comparing two data sets is defined as

(5) $$\text{C}.\text{C}.=R=\frac{\sum_{i=1}^{n}{\left(f_{i}-\overset{̅}{f}\right)}_{t}{\left(f_{i}-\overset{̅}{f}\right)}_{e}}{\sqrt{\sum_{i=1}^{n}{\left(f_{i}-\overset{̅}{f}\right)}_{t}^{2}}\sqrt{\sum_{i=1}^{n}{\left(f_{i}-\overset{̅}{f}\right)}_{e}^{2}}},$$

where the subscripts *t* and *e* denote theoretical and experimental values, respectively [63].

After *c*_1_ and *c*_3_ parameters were generated from curve fits to the experimental data, acellular constants were compared to the native constants with the baseline parameter of “percent change.” The concept of “percent change” was used rather than absolute change because each paired sample set is unique with respect to the site along the tapering aortic length from which it was taken. This approach allows the changes observed in both proximal and distal arterial sections undergoing the same decellularization protocol to be combined in a single data set.

Native samples were used as controls for each set and the percentage change of acellular constants was analyzed. This approach sets the native constants to 100% (unity):

(6) $$\left(\text{\%}\Delta c_{i}\right)=\frac{\Delta c_{i}}{{\left(\Delta c_{i}\right)}_{\text{native}}}\times 100,\;i=1,3.$$

#### 2.3.3. Statistical Methods

Paired, two-tailed *t*-test analysis was used to assess the statistical significance of percent change for acellular samples based on *c*_1_ and *c*_3_ parameters. The null hypothesis is that no difference exists between mechanical properties of native and acellular tissue, with *P* < .05 considered significant and reason to reject the null hypothesis. Two criteria, ranked in order of importance, determine the effectiveness of a decellularization protocol: (i) a narrow confidence interval (±15%) and (ii) no statistical significance among differences in Yeoh coefficients (*P* > .05). More information and background on the statistical methods used are given in the appendix.

## 3. Results

### 3.1. Optical Microscopy Results

Native arterial tissue was stained as a comparison for determining the extent to which vascular smooth muscle cells were removed from the tissue by the decellularization treatment. Protocols A and B do not effectively decellularize the arterial tissue, as blue-stained nuclei appeared in the H&E-stained sections much the same as they appeared in the native tissue section. Protocol C is the only protocol of the three that effectively decellularizes the tissue, and mechanical data was not included in the analysis if the histological samples showed incomplete decellularization. 

Native vascular smooth muscle cell density was ~4600 cells/mm^2^; for Protocol A, ~4500 cells/mm^2^, for Protocol B, ~3800 cells/mm^2^, and no cells were detected after treatment with Protocol C.

### 3.2. Mechanical Testing Result

**Figure 4:**
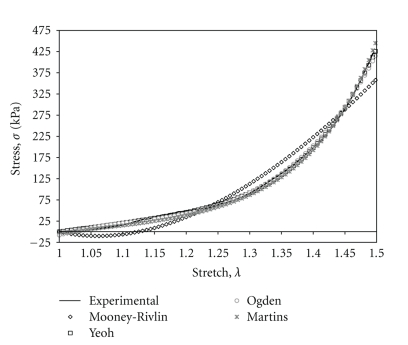
Experimental data plotted against curve fits to nonlinear material models.

For comparison between the exponential Mooney-Rivlin, Ogden, and Martins models and the general form of the Yeoh model, curve fits are plotted against representative experimental data for a longitudinal strip sample loaded in tension up to a stretch ratio of 1.50 (Figure 4). 

**Figure 5:**
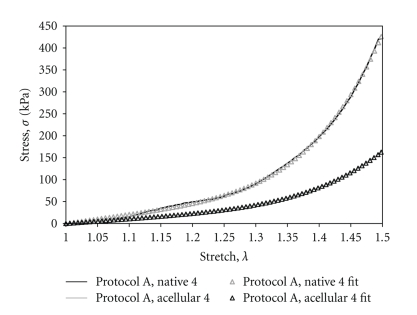
A representative plot of a paired data set and corresponding curve fits (4th load curve).

**Figure 6:**
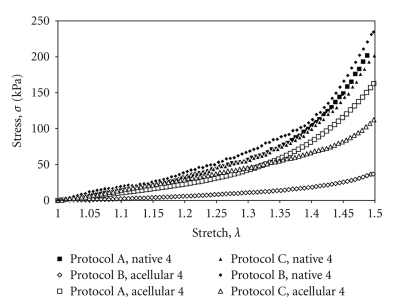
Typical final experimental load curves for native and acellular samples across all decellularization protocols tested.

Paired data sets were fit to the Yeoh material model (Figures 5 and 6). Data for *c*_1_ and *c*_3_ for each protocol, including mean, *P*-value, and standard deviation (SD) are presented in Tables 1 and 2. Incremental modulus values, which scale with the *c*_3_ parameter of the modified Yeoh curve fit equation, are evaluated at *λ* = 1.49 and are included in the extended data tables: Tables 3, 4, and 5. Past studies have identified the axial *in vivo* stretch ratio to be between *λ* = 1.40 and *λ* = 1.50 [49, 50]. 

For parameter *c*_1_, Protocol A exhibits a very wide confidence interval [10.0% ± 26.5%] which envelopes the upper and lower confidence limits of Protocol C. Protocol B [−19.1% ± 16.5%] exhibits a wider confidence interval than Protocol C [13.9% ± 13.9%]. Protocol C has the narrowest confidence interval of the three protocols, and changes are not considered statistically significant.

For parameter *c*_3_, Protocol A [−27.9% ± 65.3%] and Protocol B [8.5% ± 54.9%] do not show statistical significance, but both protocols exhibit significantly wider confidence limits than Protocol C [−39.1% ± 27.7%]. Even if Protocol C produces statistically different results (*P* = .03), the tissue's “hyperelastic” behavior changes more predictably in contrast to the effects of other protocols.

## 4. Discussion

Xenografts and allografts may be used as alternatives to stiff biomaterial implants such as Dacron and ePTFE, but most current studies have overlooked compliance in the physiological range, have flaws in experimental methodology that affect reported results, or have not directly compared the mechanics of similar native and acellular tissues. Changes in mechanical properties may indicate degradation of the extracellular matrix that would ultimately cause vessel thrombosis as a result of local injury or trigger intimal hyperplasia as a result of altered hemodynamics. Such changes were investigated by analyzing differences between the mechanical behavior of native and decellularized porcine descending aorta. Curve fits to experimental data (*R* > 0.992) were used to determine parameters for the Yeoh material model. Changes in elastic and strain-hardening material response could be inferred by changes in the model parameters, allowing a quantitative comparison between the mechanics of acellular and native tissues. The ultimate goal of this study is to identify a decellularization procedure that either minimally or predictably alters the mechanical behavior of decellularized aorta at *in vivo* stretch ratios.

Drawing on the statistical analysis of the curve fit results, if a particular decellularization protocol has narrow confidence limits (±15%) and no statistical significance among sample differences (*P* > .05), the protocol does not significantly affect the properties of the arterial tissue. With respect to these standards for evaluating protocols based on curve fit parameters *c*_1_ and *c*_3_, tissue samples treated with Protocol A and Protocol B resulted in the highest variability in mechanical behavior, evidenced by large standard deviations, and therefore large confidence intervals, in comparison to Protocol C. Based on this statistical analysis, Protocol C is expected to decrease elastic compliance and increase hyperelastic compliance. Though Protocol C causes changes in mechanical behavior which are statistically significant, its narrow confidence interval, in comparison to other protocols, may indicate these changes are repeatable. Given these conditions, it is practical to ignore the statistical significance of Protocol C when determining the most efficacious decellularization protocol.

Based upon the histological results, only Protocol C effectively lysed all vascular smooth muscle cells from the extracellular matrix. After the initial results for Protocol A and Protocol B tested positive for nuclei, the protocols were repeated with seven times the original solution-to-tissue volumetric ratio (222 : 1 increased from 30 : 1). Even at this high volumetric ratio, decellularization did not occur. 

Other biomechanical studies tested the effects of TritonX-100/EDTA, 0.05% SDS, and 0.1% SDS treatments on porcine artery with similar results. Dahl et al. used DNA quantification tests to show that a TritonX-100/EDTA protocol (Protocol A without RNase A) was ineffective at removing cells from intima-media vessels, while a 0.05% SDS treatment was effective at cell removal [37]. The same study showed decreased compliance of decellularized vessels, though only rupture properties were statistically compared. Additionally, since there were no significant differences in collagen quantification between native and decellularized vessels, collagen denaturation was ruled out as a contributing factor to alterations in mechanical compliance. In light of these results, Dahl et al. posited that an alteration in vessel compliance for the 0.05% SDS treatment was caused by the absence of vascular smooth muscle cells [37]. This explanation is incomplete at best, since the TritonX-100/EDTA and SDS-treated vessels in their study display similar mechanical behavior, even though the TritonX-100/EDTA treatment did not decellularize the tissue. Similarly, Roy et al. used a 0.1% SDS treatment in their biomechanical study and found that the treated vessels were effectively decellularized but had a pronounced decrease in mechanical compliance under inflation loadings [48]. However, Roy et al. attribute the decrease in decellularized vessels' distensibility to the early engagement of collagen fibers at low pressures [48].

Williams et al. compared the tensile properties of native and decellularized rabbit carotid specimens, employing a decellularization protocol using trypsin and ammonium hydroxide [58]. Small-angle light scattering was used to compare and relate native and acellular vessel fiber kinematics to mechanical properties. They found that the collagen fiber orientation was significantly disrupted by the decellularization process. Their decellularized arteries displayed a much greater degree of stiffness than the uniaxially-tested strips of [37] and our own study, though [37] mechanically tested vessels without an adventitial layer. Williams et al. explained that the increased stiffness occurred in decellularized arteries as the randomized fibers were recruited in the direction of applied strain [58].

Based on our results and those of [37], it appears that TritonX-100/EDTA treatments are less effective at lysing cells from porcine artery than SDS-based treatments. Both the TritonX-100/EDTA and SDS-based treatments may decrease compliance of vessels. However, the changes in compliance following the selected TritonX-100/EDTA or SDS-based treatments were significantly less than the observed changes in compliance following treatments utilizing trypsin and ammonium hydroxide. The decrease in compliance may be attributed to fiber recruitment in the direction of applied strain or collagen denaturation as a result of the chemical treatments. Though the porosity of decellularized segments limited Williams et al. to uniaxial mechanical testing [58], it would be of interest to see if changes resulting from their trypsin/ammonium-hydroxide protocol are as dramatic during inflation testing, which would constrain randomized fibers from rotating into the direction of applied strain. 

As a distinguishing aspect of this study, vessels treated with the selected protocols have been reported to be successfully decellularized and recellularized [27, 34, 60]. Decellularization methods utilizing trypsin were not considered because of reports of incomplete decellularization at short (24 h) incubation times [64] or damage to the extracellular matrix of vascular tissue at longer incubation times [33, 34, 58] unless the tissue is pretreated [65]. McFetridge et al. showed that it is possible to stabilize the extracellular matrix against excessive damage with a 75% ethanol pretreatment [65]; because subsequent stages included crosslinking, this protocol was not included among the enzymatic-detergent cell extraction protocols tested.

 Our results show that a TritonX-100/deoxycholic-acid treatment originally proposed by Rieder et al. for decellularization of porcine aortic valves [34], effectively decellularizes porcine descending aorta and induces minimal changes in mechanical properties of porcine arterial tissue at stretch ratios between 1.00 and 1.50. These results, along with those of Murase et al. [38], suggest that deoxycholic acid treatments effectively preserve the native mechanical properties and mechanical integrity of the aortic extracellular matrix while lysing cellular components. 

## Figures and Tables

**Table 1:** Breakdown of number of aortas used per tested protocol and the number of samples derived from each aortic length.

	Number of aortas	Number of samples (samples per aorta)
Protocol A	8	10 (1,1,1,1,1,1,1,3)
Protocol B	7	10 (1,1,1,1,2,2,2)
Protocol C	7	10 (1,1,1,1,2,2,2)

**Table 2:** Comparison of statistics for *c*_1_ among decellularization protocols. Italics indicate statistical significance (*P* < .05). Values shown as percentages.

	*μ* (90% C.L.)	SD	*P*
Protocol A (*n* = 10)	10.0 (26.5)	51.6	0.51
Protocol B (*n* = 10)	−19.1 (16.5)	28.7	0.06
Protocol C (*n* = 10)	13.9 (13.9)	24.2	0.10

**Table 3:** Comparison of statistics for *c*_3_ among decellularization protocols. Italics indicate statistical significance (*P* < .05). Values shown as percentages.

	*μ* (90% C.L.)	SD	*P*
Protocol A (*n* = 10)	−27.9 (65.3)	125.2	0.46
Protocol B (*n* = 10)	8.5 (54.9)	95.9	0.78
Protocol C (*n* = 10)	−39.1 (27.7)	48.2	*0.03*

**Table 4:** Extended data for Protocol A.

Sample	Native			Acellular		
	*c*_1_	*c*_3_	(*d* *θ*/*d* *λ*)|_*λ*=1.49_ (kPa)	*c*_1_	*c*_3_	(*d* *θ*/*d* *λ*)|_*λ*=1.49_ (kPa)
1	20.8	18.2	681	30.6	19.6	797
2	22.5	8.3	404	23.3	32.3	1112
3	20.0	61.1	1949	15.1	21.4	725
4	34.3	99.9	3191	17.8	33.5	1111
5	29.0	72.4	2402	23.5	27.4	987
6	31.3	42.2	1462	22.5	12.4	515
7	20.8	51.8	1711	42.7	10.7	616
8	29.3	45.0	1405	43.0	13.6	688
9	45.8	75.4	3084	41.9	11.9	840
10	30.8	67.7	3314	38.9	15.3	901

**Table 5:** Extended data for Protocol B.

Sample	Native			Acellular		
	*c*_1_	*c*_3_	(*d* *θ*/*d* *λ*)|_*λ*=1.49_ (kPa)	*c*_1_	*c*_3_	(*d* *θ*/*d* *λ*)|_*λ*=1.49_ (kPa)
1	31.1	22.1	837	25.8	67.0	1160
2	41.5	33.5	1253	30.6	15.9	639
3	25.4	24.7	903	19.5	38.6	1265
4	28.5	43.6	1486	4.6	6.7	229
5	42.4	112.5	3354	23.6	85.3	2708
6	30.9	22.6	890	30.3	75.6	2443
7	36.6	54.5	1921	30.5	22.7	885
8	27.8	22.8	856	31.9	25.8	995
9	21.1	41.2	1356	22.9	45.7	1527
10	20.0	60.3	1895	19.7	12.1	495

**Table 6:** Extended data for Protocol C.

Sample	Native			Acellular		
	*c*_1_	*c*_3_	(*d* *θ*/*d* *λ*)|_*λ*=1.49_ (kPa)	*c*_1_	*c*_3_	(*d* *θ*/*d* *λ*)|_*λ*=1.49_ (kPa)
1	13.6	46.5	1473	22.0	29.3	1044
2	20.1	34.3	1155	17.9	25.9	906
3	25.9	48.9	1667	32.3	34.0	1238
4	24.2	37.9	1278	22.6	11.3	504
5	29.8	46.0	1561	30.4	16.0	679
6	30.2	100.5	3196	29.7	37.8	1337
7	31.3	63.3	2097	36.5	17.8	784
8	16.9	96.5	2956	24.5	56.3	1796
9	27.7	103.7	3243	32.4	25.1	960
10	27.6	30.8	1097	25.4	57.8	1874

## Appendix

### Statistical Analysis Details

The resulting changes in acellular samples were analyzed with a two-tailed paired student's *t*-test analysis, since the data is expected to approximate a normal distribution. The hypotheses for this test were

(A.1) $$H_{0}\text{:}{\left(\%\Delta c_{x}\right)}_{\text{native}}-{\left(\%\Delta c_{x}\right)}_{\text{acellular}}=0,$$

(A.2) $$H_{1}\text{:}{\left(\%\Delta c_{x}\right)}_{\text{native}}-{\left(\%\Delta c_{x}\right)}_{\text{acellular}}\neq 0$$

for *x* = 1 and *x* = 3.

The result of each *t*-test is represented by a *t*-statistic, which is the number of “standard error of mean difference” intervals along a *t*-distribution between the mean and zero. The probability *P* of the null hypothesis *H*_0_ being true is calculated as a relative measure between the observed and critical *t*-statistics. If *P* < .05, zero does not lie within the 90% confidence interval

(A.3) $${\text{C}.\text{I}.}_{90\%}=\mu \pm t_{\alpha =0.05}*\text{SE}\left(\overset{̅}{d}\right),$$

and the null hypothesis (A.1) can be rejected. Note that for paired sample *t*-testing, the critical *t*-value has (*n* − 1) degrees of freedom. In (A.3), *μ* is the mean of the sample set, *t*_*α*=0.05_ is the critical *t*-statistic, and $\text{SE}\left(\overset{̅}{d}\right)$ represents the “standard error of the mean differences.” The standard error of the mean differences can be calculated from the standard deviation (SD) and number of samples *n*, as

(A.4) $$\text{SE}\left(\overset{̅}{d}\right)=\frac{\left(\text{SD}\right)}{\sqrt{n}}.$$

The effectiveness of a decellularization protocol will be evaluated based on the range of its confidence interval and on statistical significance of the differences in native and acellular sample populations. Ideally, a protocol's confidence interval should be narrow (±15%) and changes in the fit parameters should not be statistically significant.
